# Improving the Policy Utility of Cause of Death Statistics in Sri Lanka: An Empirical Investigation of Causes of Out-of-Hospital Deaths Using Automated Verbal Autopsy Methods

**DOI:** 10.3389/fpubh.2021.591237

**Published:** 2021-05-26

**Authors:** Lene Mikkelsen, Sunil de Alwis, Sridharan Sathasivam, Vindya Kumarapeli, Ajith Tennakoon, Palitha Karunapema, Kapila Jayaratne, Rajitha Jayasuriya, Saman Gamage, Roshan Hewapathirana, Rangana Wadugedara, Manoj Dissanayake, Chamika H. Senanayake, Pasyodun Koralage Buddhika Mahesh, Deirdre McLaughlin, Alan D. Lopez

**Affiliations:** ^1^Melbourne School of Population and Global Health, University of Melbourne, Parkville, VIC, Australia; ^2^Ministry of Health, Nutrition and Indigenous Medicine, Colombo, Sri Lanka; ^3^Melbourne School of Population and Global Health, The University of Melbourne, Carlton, VIC, Australia; ^4^Faculty of Medicine, University of Colombo, Colombo, Sri Lanka; ^5^University of Washington, Seattle, WA, United States

**Keywords:** verbal autopsy, SmartVA, causes of death, Sri Lanka, out-of-hospital deaths, home deaths, cause specific mortality fractions

## Abstract

**Background:** Setting public health policies and effectively monitoring the impact of health interventions requires accurate, timely and complete cause of death (CoD) data for populations. In Sri Lanka, almost half of all deaths occur outside hospitals, with questionable diagnostic accuracy, thus limiting their information content for policy.

**Objectives:** To ascertain whether SmartVA is applicable in improving the specificity of cause of death data for out-of-hospital deaths in Sri Lanka, and hence enhance the value of these routinely collected data for informing public policy debates.

**Methods:** SmartVA was applied to 2610 VAs collected between January 2017 and March 2019 in 22 health-unit-areas clustered in six districts. Around 350 community-health-workers and 50 supervisory-staffs were trained. The resulting distribution of Cause-Specific-Mortality-Fractions (CSMFs) was compared to data from the Registrar-General's-Department (RGD) for out-of-hospital deaths for the same areas, and to the Global-Burden-of-Disease (GBD) estimates for Sri Lanka.

**Results:** Using SmartVA, for only 15% of deaths could a specific-cause not be assigned, compared with around 40% of out-of-hospital deaths currently assigned garbage codes with “very high” or “high” severity. Stroke (M: 31.6%, F: 35.4%), Ischaemic Heart Disease (M: 13.5%, F: 13.0%) and Chronic Respiratory Diseases (M: 15.4%, F: 10.8%) were identified as the three leading causes of home deaths, consistent with the ranking of GBD-Study for Sri Lanka for all deaths, but with a notably higher CSMF for stroke.

**Conclusions:** SmartVA showed greater diagnostic specificity, applicability, acceptability in the Sri Lankan context. Policy formulation in Sri Lanka would benefit substantially with national-wide implementation of VAs.

## Introduction

A well-functioning Civil Registration and Vital Statistics (CRVS) system is fundamental to support the sustainable development of a country. Completeness of vital events registration, as well as the diagnostic quality of mortality data, are the two most important factors determining the proper functioning of CRVS systems (1). Worldwide, only one in four countries have well-functioning death registration systems, primarily in Europe, North America and Australasia (1, 2). Given their critical role in supporting health and development strategies in countries, strengthening CRVS systems has become an urgent policy priority, supported by new and significant investments from philanthropy and other development partners (3).

Cause of death (CoD) information is arguably the most essential component of a CRVS system from a health policy perspective (3, 4). However, globally, only about one-third of all deaths are assigned a cause that is sufficiently specific for informing policy (5). Each year, of the roughly 25 million deaths that are reported by vital registration systems in countries, about 7 million are assigned causes which are of limited use for guiding public health policy and planning, collectively known as “garbage codes” (6–8). Global initiatives such as the Bloomberg Philanthropies Data for Health Initiative (BD4H) have been established to strengthen CRVS systems in lower-and-middle-income countries (LMICs), focussing in particular on improving data collection practices and critical data analysis skills (9, 10). In order to more reliably diagnose the causes of community deaths, an automated diagnostic tool known as “SmartVA Automated Verbal Autopsy” has been trialed in several countries, including Sri Lanka (11).

SmartVA is a method for estimating population-level cause of death patterns. From the information obtained by a trained interviewer from the caretaker of the deceased person, computer algorithms can diagnose the most probable cause of death based on pattern recognition in the data. The interview covers the circumstances, signs and symptoms, and health care seeking behavior of the decedent during the terminal stage leading to death.

Automated VA is the only cost-effective alternative for generating reliable information on cause of death patterns in populations where the medical certification of CoD is not feasible (4). The value of this methodology in improving the evidence base available to countries for policy formulation and evaluation has been recognized by global health development agencies, including the World Health Organization (12).

Validation studies have demonstrated that recall accuracy for symptoms has limited effect on the diagnostic performance of VA up until about 12 months after death (13). Capturing data electronically also reduces data collection time, and data errors (14). The Tariff-method, one of the computer-based algorithms available for the analysis of VA data, has been incorporated into the SmartVA platform, and is the most intuitive and easily understood of the various diagnostic methods available (15). Comparative analyses with physician-assisted diagnosis of VA data have demonstrated the reliability and practicality of automated VA methods for the generation of population level CoD statistics cheaply, rapidly and in a standardized fashion (4). The Tariff diagnostic algorithm yields diagnoses for 33 mutually exclusive and exhaustive common causes of adult deaths, typically covering about 80-90% of the most common causes of death in LMICs (16, 17).

In some cases, the “signal-to-noise” ratio from the VA interview is too poor to confidently diagnose the most probable cause of death. In these cases, typically 10–15% of all VAs, the cause of death is “undetermined”. These deaths can then be reallocated to one of the 33 defined causes based on knowledge about the epidemiology of the population and the comparative ability of the algorithm to identify specific causes (18). Following the re-distribution of undetermined causes, all deaths are assigned a cause and aggregated to yield population -level cause-specific mortality fractions (CSMFs). More detail on the development of the Tariff diagnostic algorithm, on the shortened VA questionnaire, and the development and performance of the SmartVA package, can be found elsewhere (15, 18–20).

As part of the D4H Initiative, the Government of Sri Lanka expressed interest in the potential application of automated VA methods for routinely diagnosing out-of-hospital deaths as a cost-effective alternative to the current practice of assigning a cause of death by non-health officers, often leading to poorly defined causes of death of limited usefulness for policy and planning in the health sector. In order to establish the validity of the methodology, and whether or not it was an improvement on current practice, we compared CSMFs from the application of SmartVA to those from current methods being applied for the determination of CoD of community deaths, for both the Sinhalese and the Tamil-speaking populations. Our primary aim was to ascertain whether SmartVA is applicable in reducing undetermined cause of death data for out-of-hospital deaths in Sri Lanka, and hence enhance the value of these routinely collected data for informing public policy debates.

## Methods

### Study Population

Approximately one-half of the 130,000 deaths which occur each year in Sri Lanka take place outside a healthcare facility (21). Under the current CRVS system in Sri Lanka, an appointed divisional-level officer without medical training i.e., the Birth and Death Registrar, assigns a cause to these out-of-hospital deaths based on a brief questionnaire asked of the family of the deceased (22, 23) (Supplementary Figure 1). In essence, this system is designed more to ensure that all deaths are registered and given a cause of death than to ensure that an accurate cause has been assigned.

Improving the usability of the CoD data for these out-of-hospital deaths has long been a priority for policy makers and health planners in Sri Lanka. To oversee the trial of SmartVA in Sri Lanka under the D4H Initiative, a Technical Working Group (TWG) on VA was established comprising key local stakeholders (22) who guided the implementation of the study. The main task of the TWG on VA was to advise on study design, including the selection of administrative areas and districts for collecting the interviews for the different phases of the study, as described in Supplementary Figure 2.

The main phase of data collection was preceded by a pilot phase applied to a convenience sample of approximately 300 adult deaths. The main purpose of the pilot was to assess the feasibility of the tablet-based verbal autopsy methodology. The interviews were carried out using Public Health Midwives (PHM), most of whom had not previously been exposed to tablet technology. They did this within the context of their existing domiciliary care-based model using the two local languages. Minor linguistic fine-tuning of the questionnaire was done following this phase.

Subsequently the main-phase of data collection was conducted in 6 districts clustered in the Western/North Western, Central and the Northern parts of Sri Lanka (Supplementary Figure 3). One of the districts (Colombo) is further divided into two health administration areas; a Regional Directorate area (i.e., Colombo-RDHS) and a Municipal Council area (i.e., Colombo-CMC). All six divisions of the Colombo-CMC region and two to three areas from other regions were selected for the study based on their representativeness within the district as well as the feasibility of obtaining sufficient cases (Supplementary Table 1).

Based on the finding of a relatively high CSMF for stroke among the Sinhalese speaking population sample, modifications were made to the terminology used for some questions relating to possible stroke symptoms, which had arisen when translating the questionnaire. Subsequently, an extended phase was conducted in a sample of deaths in Colombo-CMC area based on this modification.

### Instrument and Data Collection

The SMART-VA verbal autopsy questionnaire was used to collect data (19), using the validated and item-reduced shortened version of the Population Health Metrics Research Consortium (PHMRC) data instrument (16, 24, 25). The questionnaire (Sinhalese, Tamil or English) was incorporated into a Smart-tablet using an Open Data Kit application. The questionnaire included 4 main modules;

- Module 1- General module: containing details of the deceased, respondent, characteristics of the death and the interviewer.
- Module 2- Adult module.
- Module 3- Child module.
- Module 4- Neonatal module.

Case definitions were used to divide the deceased into three strata (see Supplementary Table 2).

After completing Module 1, the relevant module for each death was automatically selected by the software application based on the age of the deceased. The completed questionnaires were saved in the tablets and transmitted to a central database following verification by a trained supervisor.

The TWG on VA, in consultation with the Director General of Health Services, concluded that the most suitable interviewers for data collection would be the PHM, who are attached to the field health institutions of the Ministry of Health and Indigenous Services (MOH). The PHM are a well-recognized field-staff category in Sri Lanka involved in the delivery of community health services mainly related to the provision of maternal and child health (26). A series of well-structured training programmes was conducted in all selected districts. Each workshop was attended by an average of 20 PHM. Training sessions included practical sessions as well as mock interviews. In addition, ethical considerations and the development of “soft-skills” (i.e., listening skills, empathizing, non-verbal communication etc.) were also included in the curriculum as well as training in using the tablets. A field-based practical session which included each PHM conducting the Smart-VA interview under supervision, was incorporated into all workshops. The Medical Officer of Health, a Public Health Nursing Sister or a Supervisory PHM were also given a 1-day training intended for data collection supervisors.

### Ensuring Sustainability

The collection of the VAs was conducted by the MOH and its staff and overseen by the TWG on VA who guided decision making on all logistical as well as administrative aspects, collaborating with University of Melbourne staff for technical inputs. The Registrar General's Department and the Health Information Society of Sri Lanka (HISSL) also assisted with implementation, by providing information about the households that had registered deaths.

The supervisory officers from the MOH were instructed in the resolution of potential issues related to tablets and transfer of data; more complex information-technology (IT) problems arising from the data collection were resolved by health-information experts at HISSL. Operational instructions were negotiated with the district level supervisory officers regarding the involvement of the PHM in data collection without interrupting their routine duties. Approximately 350 PHM were trained and provided with a Smart-Tablet device. In addition, around 50 supervisory officers were trained to oversee data collection and provide quality control checks on the work of the interviewers. The collection of the data proceeded as a service-requirement of the Ministry of Health. Two evaluation workshops were held with data collectors to discuss any challenges experienced in implementing the questionnaire.

### Data Analysis

The data were processed by the MOH through the “SmartVA-Analyze” software after duplicate cases were identified and removed, and data were cleaned. Since the number of neonatal and child deaths were insufficient for meaningful interpretation, our analysis is limited to adult deaths (which in any case comprise the vast majority of deaths in the country).

In order to assess the plausibility of the CSMFs derived from application of the SmartVA methodology, the results were compared with estimates from the Global Burden of Disease (GBD) Study for Sri Lanka (27). While this is not strictly a comparable population, since the GBD also includes deaths in hospitals in Sri Lanka, it is the only available data on corrected (for ill-defined causes) CSMFs for Sri Lanka. Since the full list of diagnoses in the GBD cause of death classification differs from that of SmartVA, comparisons of leading causes of death with the GBD were done by mapping the GBD cause to the list of VA causes. The other comparator data set used was home deaths from the sampled districts identified in the latest available (2014) annual data set from the RG Department. These data were entered into the ANACONDA software (28) to identify the leading causes of home deaths.

## Results

It was demonstrated that despite having little or no experience with tablets, it was possible to train 350 data collectors to competently carry out interviews with tablets and having these uploaded to a central server in the Ministry without any significant IT problems being reported. In the two evaluation workshops, no refusals were reported and the additional time burden for the PHMs was judged acceptable.

A total of 2,623 VAs were performed for adult deaths, and of these, 2,610 cases (1,250 male, 1,358 female, 2 sex unknown) met the analytical pre-requisites for SmartVA-Analyze. The median (IQR) age at death in the adult sample was 74 (64 to 82) and 79 (70 to 86) years for males and females, respectively. No respondents refused the VA interview.

The age-at-death distribution is given in Table 1. For comparison, the age-distribution of out-of-hospital deaths for corresponding areas from the Registrar General-(RG) data for 2014 is also shown, along with the estimated age-distribution of deaths for Sri Lanka in the GBD Study.

**Table 1:** Age distribution of deaths for the VA sample and for out-of-hospital deaths in corresponding areas recorded in the RG data (2014) and estimated by the GBD (2017).

**Age group**	**Total** ***N*** **(%)**		
	**SmartVA**	**RG**	**GBD**
12–14^*^	2 (0.1)	5 (0.1)	357 (0.3)
15–19	8 (0.3)	23 (0.5)	905 (0.7)
20–24	2 (0.1)	35 (0.7)	1,452 (1.2)
25–29	50 (1.9)	38 (0.8)	1,444 (1.2)
30–34	3 (0.1)	46 (0.9)	1,647 (1.3)
35–39	18 (0.7)	39 (0.8)	2,211 (1.8)
40–44	25 (1.0)	76 (1.6)	2,944 (2.4)
45–49	29 (1.1)	105 (2.1)	4,304 (3.5)
50–54	67 (2.6)	211 (4.3)	6,176 (5.0)
55–59	130 (5.0)	231 (4.7)	8,445 (6.9)
60–64	179 (6.9)	389 (7.9)	1,1215 (9.1)
65–69	288 (11.0)	521 (10.6)	14,400 (11.7)
70–74	330 (12.6)	690 (14.1)	15,852 (12.9)
75–79	427 (16.4)	724 (14.8)	15,333 (12.4)
80–84	413 (15.8)	734 (15.0)	15,582 (12.6)
85+	625 (23.9)	1,030 (21.0)	20,947 (17.0)
Unknown	14 (0.5)	1 (0.0)	0 (0)
Total^**^	2,610 (100.0)	4,898 (100.0)	123,213(100.0)

* *For RG and GBD data, this age category was 10–14 years*.

** *For 2 deaths, sex category is missing in VA*.

Of the 2,610 adult VAs, a cause of death could not be determined for 362 (13.9%), more so for females (16.9%) than for males (10.5%). Table 2 shows the CSMFs for adult deaths in the sample after re-distributing the undetermined causes according to the algorithm proposed by Serina et al. (18). Stroke, chronic respiratory diseases, ischemic heart disease, and diabetes were identified as the leading causes of death in this sample of home deaths, followed by other non-communicable diseases, collectively accounting for almost three-quarters (72%) of all adult deaths.

**Table 2:** Cause specific mortality fractions (after-redistribution of undetermined cases), adult deaths, Sri Lanka, 2017–18.

**Cause**	**Total (%)**	**Male (%)**	**Female (%)**
Stroke	33.9	31.6	35.4
Ischemic heart disease	13.5	13.5	13.0
Chronic respiratory	13.1	15.4	10.8
Diabetes	8.2	6.7	9.5
Other non-communicable diseases	3.3	3.1	3.4
Chronic kidney disease	3.1	4.2	2.0
Falls	3.0	1.9	3.9
Lung cancer	2.4	4.0	0.9
Diarrhea/dysentery	2.2	1.7	2.5
Pneumonia	2.2	2.1	2.2
Esophageal cancer	1.9	2.6	1.2
Other injuries	1.5	1.3	1.5
Prostate cancer	1.5	3.0	0.0
Cervical cancer	1.4	0.0	2.6
Leukemia/lymphomas	1.1	1.2	0.9
Breast cancer	1.0	0.0	2.0
Stomach cancer	1.0	0.9	1.1
Cirrhosis	0.9	1.4	0.5
Other cancers	0.9	0.9	1.0
TB	0.8	1.1	0.6
Other infectious diseases	0.7	0.5	0.8
Other cardiovascular diseases	0.5	0.3	0.6
Poisonings	0.4	0.3	0.5
Colorectal cancer	0.3	0.3	0.4
Homicide	0.3	0.2	0.3
Road traffic	0.3	0.3	0.2
Suicide	0.3	0.3	0.2
Drowning	0.2	0.2	0.2
Fires	0.1	0.1	0.2
Bite of venomous animal	0.0	0.0	0.0
Maternal	0.0	0.0	0.1

To assess the plausibility of the age-patterns of causes of death in the sample, Supplementary Figures 4A,B compares the age-distribution of the three leading causes of death diagnosed by SmartVA with what the GBD reports for the same causes.

**Figure 1:**
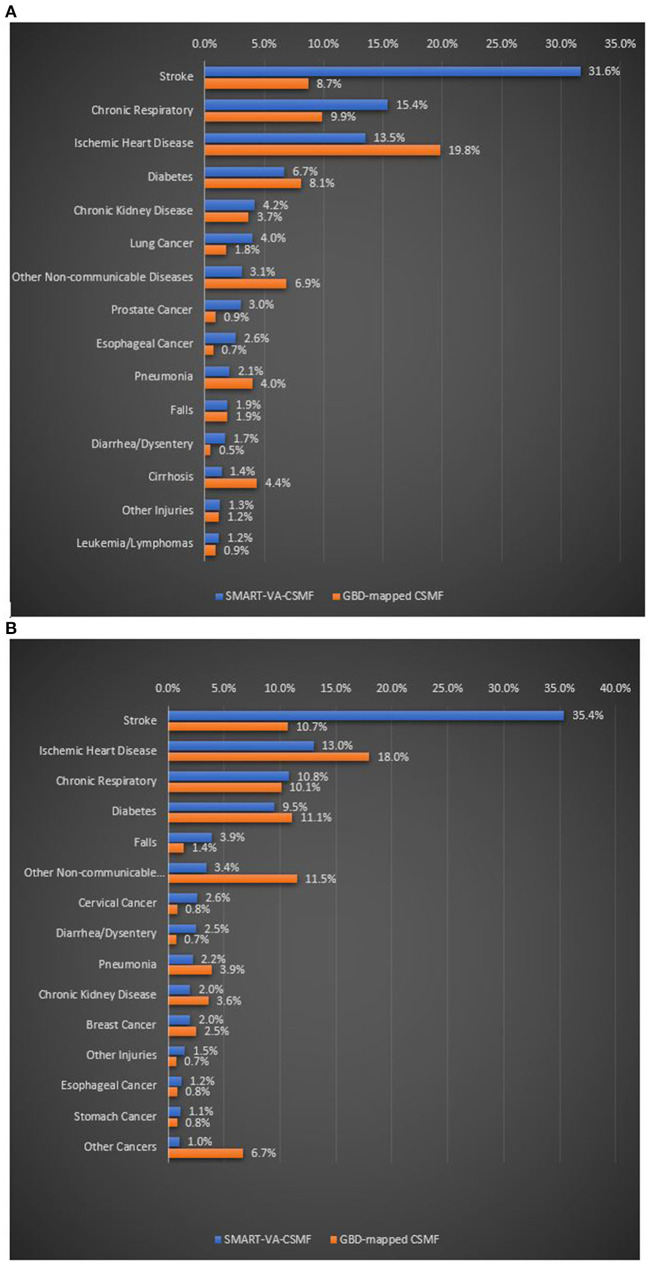
**(A)** Leading 15 CSMFs for adult males estimated by SmartVA and the GBD, Sri Lanka, 2017–18. **(B)** Leading 15 CSMFs for adult females diagnosed by SmartVA and the GBD, Sri Lanka, 2017–18.

We also assessed the plausibility of the CSMFs from SmartVA by comparing the leading causes of death, by sex, with what has been estimated for Sri Lanka by the GBD (Figures 1A,B). While for many causes of death, the cause fractions are reasonably similar, they are substantially different for the three leading causes, Stroke, IHD and Chronic respiratory disease, more so for males. For stroke in particular, SmartVA estimates a much higher fraction of deaths than the GBD. While some of this difference undoubtedly arises due to the older age structure of out of hospital deaths in the VA sample, the differences are sufficiently large to suggest systematic under-estimation of stroke deaths by the GBD and possibly a tendency to over-diagnose stroke deaths by SmartVA. For females, stroke was also substantially higher in the VA sample than in the GBD, while the converse was true for IHD. For females, CSMFs for chronic respiratory disease (and diabetes) were similar.

The stroke: IHD mortality ratio was reversed between the two sets of estimates, being substantially >1 for the VA population, as diagnosed by SmartVA, and <1 according to the GBD. In part, this could be due to the very old age structure of the VA sample deaths. Table 3 compares the CSMFs of the 10 leading causes according to the main-language related geographical areas. CSMFs for ischemic heart disease, chronic kidney disease and pneumonia were notably higher for the (Tamil) areas of the Northern cluster compared to areas where Sinhalese was used as the main language. The fraction of deaths due to stroke was higher among the Sinhalese-speaking population. Interestingly, the stroke: IHD mortality ratio was much closer to one for the Tamil speaking areas than for the Sinhalese speaking populations.

**Table 3:** Leading causes of death (CSMFs), adults, Sri Lanka, according to main-language regions.

**Areas with Sinhalese as the**		**Areas with Tamil as the**	
**main language**		**main language**	
**Cause**	**%**	**Cause**	**%**
Stroke	37.0	Stroke	22.0
Chronic respiratory	12.4	Ischemic heart disease	19.5
Ischemic heart disease	12.4	Chronic respiratory	11.5
Diabetes	8.2	Diabetes	8.8
Other non-communicable diseases	3.3	Chronic kidney disease	6.6
Falls	3.0	Pneumonia	4.9
Chronic kidney disease	2.7	Other non-communicable diseases	3.5
Diarrhea/dysentery	2.4	Lung cancer	3.5
Lung cancer	2.2	Esophageal cancer	2.9
Esophageal cancer	1.8	Prostate cancer	2.0

**Figure 2:**
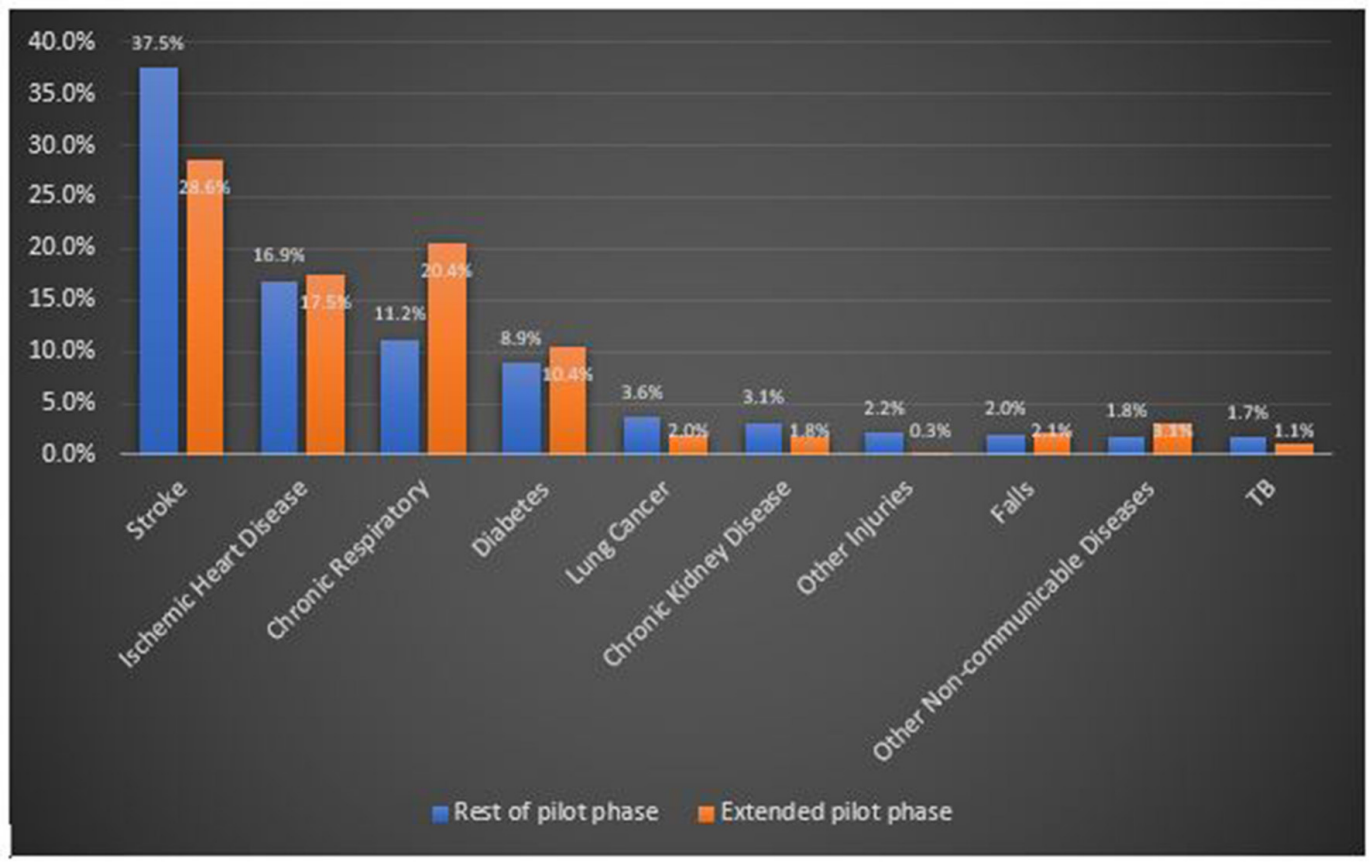
Comparison of the 10 leading causes of death diagnosed by SMARTVA in the Colombo CMC area before and after modification of the terminology used for stroke symptoms.

In order to investigate whether the wording change made when translating the questionnaire had had a significant impact on the CSMF for stroke, we compared, for the Colombo CMC population only, the CSMFs of VAs from the main phase with those collected following the slightly amended questionnaire in the extended phase, as shown in Figure 2. While stroke still remained the leading cause of death in the extended phase, the CSMF decreased substantially from 37.5 to 28.6%, between the two samples. The converse was true for chronic respiratory diseases. Although based on a relatively small sample size (250 deaths), this analysis suggests that the CSMF for stroke may have been overestimated by as much as 25–30% due to the rewording of the Sinhalese questionnaire following the pilot phase. This should be kept in mind when interpreting the results of the main study and may partially explain the difference in the CSMFs compared with the GBD.

In order to assess the policy utility of SmartVA, we calculated the amount of “garbage codes,” (i.e., codes with no or limited value for public health policy and planning) in the RG data for 2014, taken from the same geographical areas as the SmartVA sample (Table 4). Based on current practice for deaths which are not medically certified, nearly 65% of male deaths and 75% of female deaths were assigned a cause of death that has little or no policy value. In Sri Lanka, the leading “garbage codes” which severely limit the policy value of the data include senility, cardiac arrest & shock, hemi/para/quadriplegia, and heart failure. In stark contrast, only about 10% of male adult deaths, and 17% (one in six) of female deaths, many of them at very old ages, could not be diagnosed with a specific cause using SmartVA. In other words, SmartVA reduced the fraction of home death diagnoses that are of no or little value for guiding policy by about 85% for males, and 75% for females.

**Table 4:** “Garbage codes” classified according to four severity levels among out-of-hospital deaths in the VA areas.

**Severity level**	**Both sexes *N* (%)**	**Males *N* (%)**	**Females *N* (%)**
Very high^a^	1,681 (34.1)	724 (29.3)	957 (38.9)
High^b^	375 (7.6)	167 (6.8)	208 (8.5)
Medium^c^	784 (15.9)	434 (17.6)	350 (14.2)
Low^d^	629 (12.8)	301 (12.2)	328 (13.3)
Total	3,469 (70.4)	1,626 (65.8)	1,843 (74.9)

*Source: Registrar general 2014 data; Total deaths = 4,929; 2,470 males, 2,459 females*.

a *Very High (Level 1), Garbage codes with ***serious impact***: causes for which the true underlying cause of death could in fact belong to more than one broad cause group*.

b *High (Level 2), Garbage codes with ***substantial impact***: causes for which the true cause of death is likely to belong to only one or two of the three broad groups*.

c *Medium (Level 3), Garbage codes with ***important impact***: causes for which we know that the true underlying cause of death is likely to be one within the same ICD chapter*.

d *Low (Level 4), Garbage codes with ***limited impact***: in this case, the uncertainty of diagnosis of the true cause of death is likely to be confined to a single disease or injury category*.

## Discussion

Although the SmartVA methodology has been applied in several countries (11) to diagnose the leading causes of community deaths, it was particularly important to demonstrate its feasibility in Sri Lanka to improve the specificity of causes of death data for home deaths, given the relatively large fraction (nearly 50%) of all deaths that they comprise (21). Our study, conducted in diverse settings and in the two main language groups of Sri Lanka, demonstrated the feasibility and value of the methodology in identifying the most probable causes of out-of-hospital deaths in the country, suggesting a vastly different epidemiological pattern to that based on current practice. Around 40% of deaths diagnosed by the RG staff under current practice were assigned “garbage codes” classified as having “serious or substantial impact” for misguiding policy debates, compared with 14% of cases for which SmartVA could not determine the cause of death.

In order to assess the plausibility of the SmartVA findings on mortality patterns, we compared the direct evidence produced by the method with the estimates from the GBD Study, which makes extensive use of covariates to model disease and injury patterns, as well as adjustments to the data to correct for misdiagnoses (5). Given the similarity of age at death distributions across all three data sources (VA, RG and GBD) there is unlikely to be a major age-structure effect on comparisons of CSMFs between SmartVA and current practice (RG data), although the markedly higher fraction of deaths at the oldest ages in the VA sample could bias upwards CSMFs for causes such as stroke which dominate at these ages. The age patterns of mortality for each of three leading causes of death calculated from SmartVA are very similar to what might be expected based on the extensive epidemiological consistency adjustments that characterize the GBD (5), with a progressive rise in the number of deaths with advancing age. The exceptional decline in the proportion of male deaths from ischaemic heart disease after age 80 is similarly observed in the GBD estimates and would appear to be real.

Application of automated verbal autopsy methods to diagnose community deaths in Sri Lanka would likely have many other benefits for improving the information base for policy. Currently, the compilation of national-level data on out-of-hospital deaths through the civil registration system is time consuming, with the result that data are generally well-out of date; for example, as of the end of 2018 when this study was undertaken, the latest available data from the RG's Department referred to 2014. On the other hand, SmartVA being an automated procedure, provides data in real time, and does so cost-effectively (the only additional cost being the purchase of tablets and the costs for training of interviewers), and in a standardized fashion, without the need for physician involvement. In short, our study has demonstrated the feasibility and policy utility of the outputs generated from applying SmartVA routinely for all home deaths in Sri Lanka, providing much greater specificity in causes of death compared with current practice. While we have shown that automated verbal autopsy methods can help increase the policy value of mortality data for community deaths, careful validation studies should be periodically conducted to ascertain the diagnostic accuracy, not only of verbal autopsy information, but also of deaths medically certified in hospitals.

Given the advanced stage of epidemiological transition characterized by relatively low mortality in Sri Lanka, the divergences from the GBD estimates require further investigation. There are several possible reasons for this. First, the GBD estimates refer to all deaths (either at home or in hospital) and the extent to which hospital deaths might have a different cause structure than home deaths would certainly affect this comparison. This might well be the case for stroke deaths and chronic respiratory diseases, which are much more common in elderly populations. Indeed, the VA population included a much higher proportion of deaths above age 80 compared to what was estimated by the GBD, including hospital patients who return home to die. Common causes of death at these advanced ages are likely to be stroke and COPD. Further, the data sources used in the GBD covariate modeling processes for Sri Lanka might be biased toward IHD as a diagnosis over stroke, leading to artificially low CSMFs for stroke. This would require a detailed investigation of how the GBD cause of death models for Sri Lanka have been developed. Finally, the higher stroke CSMFs from SmartVA are likely to be in part artefactual, resulting from the changes in terminology applied to some of the stroke symptom questions after the pilot study. These changes may well-have led to an inflation of the stroke CSMF by about one-quarter for the Sinhalese speaking population. While this would not affect our primary conclusions about the importance of stroke as the leading cause of home deaths in Sri Lanka, it would imply stroke CSMFs that were somewhat closer to the GBD.

There are several limitations of our study that should be mentioned. Firstly, although all data collectors underwent the same training, differences in aptitude, commitment and comprehension among interviewers may have affected the comparability of the findings across population subgroups. Secondly, despite the rigorous translation procedures applied context-specific linguistics may have affected the clarity of some of the questions from the respondents' perspective, this risk was emphasized in all trainings and the data collectors were empowered to convey the expected meaning of each question in cases of uncertainty by the respondent. Finally, the difference in time period between the reference data set as collected by the BDR (2014) and the deaths diagnosed by SmartVA (2017-18) might have led to some bias in the comparisons of cause –specific fractions between the two data sources. This is unlikely to have been substantial, however, given the fact that the vast majority of deaths in Sri Lanka are attributable to non-communicable diseases, which typically change only slowly, and in the absence of any significant shocks from disease pandemics, or natural disasters, in either dataset.

## Conclusions and Recommendations

Automated verbal autopsy methods can feasibly be applied in Sri Lanka to generate much more reliable and standardized information on the causes of home deaths, and more rapidly, than current practice. We were able to generate over 2,500 VAs by training 350 data collectors from diverse areas of the country to competently carry out family interviews, typically in <30 min, using tablets, and to upload the data to a central server for analysis. Despite having little or no experience with tablets, the PHMs had no difficulty in operating them and no significant IT problems were reported.

The results obtained from the application of SmartVA in Sri Lanka, particularly the much greater diagnostic specificity of diagnoses for home deaths compared with current practice, the overall plausibility of the findings in the context of Sri Lanka's epidemiological transition, the cultural acceptability and effectiveness of the interviews, collectively support the application of SmartVA throughout Sri Lanka as routine practice to supplement current procedures in order to dramatically improve the utility of CoD data for out-of-hospital deaths. Health information systems in Sri Lanka that support policy formulation and evaluation would benefit substantially from the nationwide implementation of SmartVA, with context-based customizations to facilitate sustainability.

## Data Availability Statement

The datasets presented in this article are not readily available because these data are owned by the Ministry of Health of Sri Lanka and any application for acess to these data would need to be addressed to the Ministry of Health in Sri Lanka. Requests to access the datasets should be directed to Sridharan Sathasivam, drsri94115@gmail.com.

## Ethics Statement

Ethical approval was not provided for this study on human participants because not applicable as the investigation was implemented as a service requirement. Data were anonymized and irreversibly de-identified. The patients/participants provided their written informed consent to participate in this study.

## Author Contributions

LM, SA, SS, VK, RJ, DM, and AL conceived the design of the technical activity. AT, PK, KJ, VK, and RJ oversaw the implementation. LM, VK, RJ, RH, RW, MD, CS, and PM contributed in training of data collectors. VK, RJ, SG, RH, RW, MD, CS, and PM were involved in activities related to coordination of data collection and data cleaning. VK, RJ, MD, and PM were involved in data analysis. SA, SS, VK, AT, PK, KJ, and SG provided feedback on data analysis. LM, AL, DM, and PM contributed in drafting the initial manuscript. All authors contributed to the framework construction, results interpretation, manuscript revision, and approved the final version of the manuscript. The corresponding authors attest that all listed authors meet authorship criteria and that no others meeting the criteria have been omitted.

## Conflict of Interest

Investigators affiliated with the Melbourne School of Population and Global Health, are involved in the activities related to the Tariff method-one of the algorithms used in verbal autopsies, in its SmartVA (i.e., a Verbal Autopsy methodology) method. However, the analysis does not include a component that compare the performance of Tariff method with other algorithms. The authors declare that the research was conducted in the absence of any commercial or financial relationships that could be construed as a potential conflict of interest.
