# Supplementary material for: Improving the Policy Utility of Cause of Death Statistics in Sri Lanka: An Empirical Investigation of Causes of Out-of-Hospital Deaths Using Automated Verbal Autopsy Methods
Source: Front Public Health. 2021 May 26;9:591237. doi: 10.3389/fpubh.2021.591237 (PMC8187752; doi:10.3389/fpubh.2021.591237)
Supplement: Supplementary file 1 [file Data_Sheet_1.docx]

**Supplementary electronic materials**


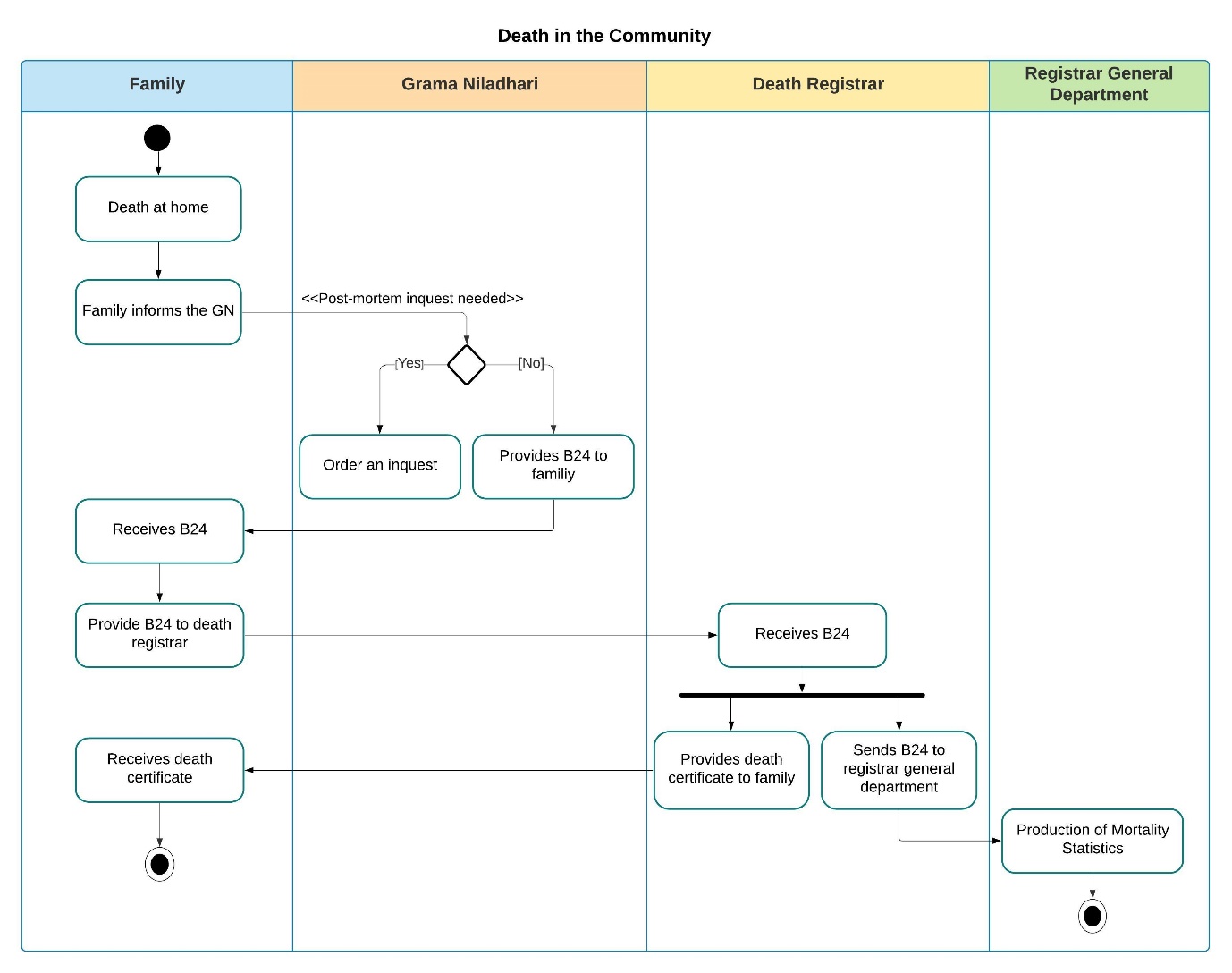


**Supplementary figure 1: Data flow for an out-of-hospital death in Sri Lanka**

| Phases | | | **Geographical locations*** | **Time duration** |
| --- | --- | --- | --- | --- |
| Pilot phase  (N=291) |  | | Colombo, Kurunegala Jaffna | From Jan to March 2017 |
|  | Main-Phase with original questionnaire in Sinhalese and Tamil languages  (N=2626) | | Colombo- CMC  Colombo-RDHS  Kurunegala  Matale  Nuwara Eliya  Jaffna  Kilinochchi | From Jan 2018 to 31^st^ March 2019 |
|  |  | Extended-phase with modified questionnaire in Sinhalese language  (N=215) | Colombo- CMC | From mid-January 2019 to 31^st^ March 2019 |

*Except Colombo-CMC all other areas correspond to the respective Regional Director of health Services (RDHS) areas

**Supplementary figure 2: Phases of Smart-VA pilot implementation in Sri Lanka, 2017-19**

**
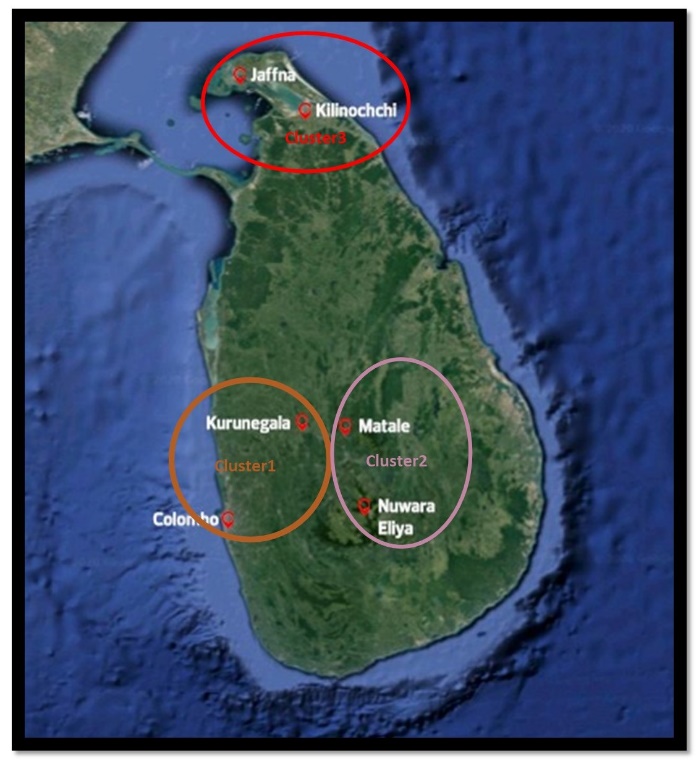
**

**Supplementary figure 3: Geographical locations of Districts in the pilot-phase implementation of SMART VA, Sri Lanka**

**Supplementary table 1: The selected MOH areas from the 6 districts**

| **Cluster** | **District/ administrative division** | **MOH areas** | **Main Language used for data collection** |
| --- | --- | --- | --- |
| 1 | Colombo-CMC | All 6 divisions | Sinahala |
|  | Colombo-RDHS | Kesbewa | Sinahala |
|  |  | Piliyandala | Sinahala |
|  |  | Ratmalana | Sinhala |
|  | Kurunegala | Rideegama | Sinahala |
|  |  | Ibbagamuwa | Sinahala |
| 2 | NuwaraEliya | Bogawanthalawa | Mixed |
|  |  | Ambagmuwa | Mixed |
|  | Matale | Dambulla | Sinahala |
|  |  | Dambulla-Municipal council | Sinhala |
| 3 | Jaffna | Kopai | Tamil |
|  |  | Nallur | Tamil |
|  |  | Uduvil | Tamil |
|  | Kilinochchi | Kandawalai | Tamil |
|  |  | Kaachchi | Tamil |
|  |  | Palai | Tamil |
|  |  | Poonekary | Tamil |

**Supplementary table 2: Case definitions and other eligibility criteria used in the pilot study**

| Stratum | Inclusion criteria | other eligibility criteria |
| --- | --- | --- |
| Adult deaths | -The deceased being 12 years or above at the time of death | Inclusion criteria:  An interview that was conducted within six weeks up to one year from the date of death  Exclusion criteria:  An inquest had been held to inquire into the cause of death |
| Child deaths | -The deceased between 29 days to 11 years at the time of death |  |
| Neonatal deaths | -The deceased dying within 28 days of birth |  |

**Supplementary figure 4a: Age distribution of the leading three causes of adult male deaths, SmartVA and the GBD**

**Supplementary figure 4b: Age distribution of the leading three causes of adult female deaths, SmartVA and the GBD**
